# Dynamic Data Visualization with Weave and Brain Choropleths

**DOI:** 10.1371/journal.pone.0139453

**Published:** 2015-09-29

**Authors:** Dianne Patterson, Thomas Hicks, Andrew Dufilie, Georges Grinstein, Elena Plante

**Affiliations:** 1 The University of Arizona, Speech, Language, and Hearing Sciences Department, Tucson, AZ, United States of America; 2 The University of Arizona, School of Information: Science, Technology, and Arts, Tucson, AZ, United States of America; 3 The University of Massachusetts Lowell, Computer Science Department, Lowell, MA, United States of America; Shenzhen Institutes of Advanced Technology, CHINA

## Abstract

This article introduces the neuroimaging community to the dynamic visualization workbench, Weave (https://www.oicweave.org/), and a set of enhancements to allow the visualization of brain maps. The enhancements comprise a set of brain choropleths and the ability to display these as stacked slices, accessible with a slider. For the first time, this allows the neuroimaging community to take advantage of the advanced tools already available for exploring geographic data. Our brain choropleths are modeled after widely used geographic maps but this mashup of brain choropleths with extant visualization software fills an important neuroinformatic niche. To date, most neuroinformatic tools have provided online databases and atlases of the brain, but not good ways to display the related data (e.g., behavioral, genetic, medical, etc). The extension of the choropleth to brain maps allows us to leverage general-purpose visualization tools for concurrent exploration of brain images and related data. Related data can be represented as a variety of tables, charts and graphs that are dynamically linked to each other and to the brain choropleths. We demonstrate that the simplified region-based analyses that underlay choropleths can provide insights into neuroimaging data comparable to those achieved by using more conventional methods. In addition, the interactive interface facilitates additional insights by allowing the user to filter, compare, and drill down into the visual representations of the data. This enhanced data visualization capability is useful during the initial phases of data analysis and the resulting visualizations provide a compelling way to publish data as an online supplement to journal articles.

## Introduction

In our highly connected world, static descriptions in journal articles are no longer the best way to share neuroimaging data or insights. The field of neuroinformatics attempts to address this problem for the neuroimaging community by promoting databases of brain images with associated data as text and static images (http://www.nitrc.org/search/?type_of_search=group&cat=313:Database), atlases that summarize article results (Neurosynth: neurosynth.org; Brede: http://neuro.imm.dtu.dk/services/brededatabase/) and other tools to view brain images interactively on the web [1]. Although these resources have improved neuroimaging reports, the coupling between brain images and the accompanying related data remains ad hoc. Even if related data is available in textual form, it is up to the researcher to download that data, prepare it for input to statistical programs or as a spreadsheet, and try to draw conclusions about its relationships to the imaging data. Static figures can supplement textual data but are problematic when used alone because the representations are difficult to reverse engineer into data. Individual researchers face similar issues in exploring their own data because the tools available for charting and graphing are entirely separate from the tools for viewing brain images.

### 1.1 Current Tools

We are not aware of any tools for the parallel exploration of brain images and related data (e.g., BOLD-derived values, genetic information, test scores). On the one hand, traditional neuroimaging tools such as FSL (http://fsl.fmrib.ox.ac.uk/fsl/fslwiki/), Afni (http://afni.nimh.nih.gov/afni/) and SPM (http://www.fil.ion.ucl.ac.uk/spm/) provide excellent preprocessing and analysis pipelines. These tools also display orthogonal brain slices and 3D reconstructions, but they are not built to represent or explore the associated data. On the other hand, standard desktop statistical tools can represent associated data locally, but don’t have a way to display brain images. Current tools fall short of our goals in two regards. First, the traditional neuroimaging tools and the desktop statistical tools are not tightly coupled. Second, they frequently lack web accessibility [1]. Tools that could provide concurrent exploration of brain images and related data would be useful for both local data exploration and data publication on the web.

### 1.2 Information Visualization

The field of information visualization is concerned with the visual representation of complex data in ways that enhance our ability to reason, understand and develop insight from the data [2]. The field of information visualization has produced a variety of web-based interactive data visualization tools and libraries in the last few years (i.e., D3: http://d3js.org/; Leaflet: http://leafletjs.com/; Weave: https://www.oicweave.org/ and others). Of these tools, Weave (WEb-based Analysis and Visualization Environment) has compelling features for our purposes. Weave is an open source, database-aware, exploratory workbench that can work as a stand-alone desktop or web application. It provides a flexible set of map and graph tools that allow layering, customization, dynamic linking, modification, and filtering of data [3]. As a result, end-users can quickly and easily navigate and filter visualizations corresponding to hundreds of different data subsets from underlying tables, and probe over visualization features to display relevant details. This flexibility facilitates information extraction [2]. Additionally, the Weave server offers the researcher granular control over the tools available for outward facing web visualizations: for example, it is possible to restrict access to the supporting data tables, the tool modification menus, and the session save and export menus. Our addition of brain choropleths and our accompanying illustrated tutorial [see “Additional Resources” at the end of this paper] facilitates the use of the Weave workbench with neuroimaging data.

### 1.3 Choropleths

A choropleth is a geographic map used to represent regional statistical information (e.g., political, socioeconomic, epidemiological) [4]. Choropleths have been used since the early 19th century [5] and continue to be used in modern information visualization tools to simplify data representation. However, the simplification inherent in such a region-based analysis comes at a cost: choropleths represent data as having sharp breaks at regional boundaries but no variation within regions. Despite (or perhaps because of) this simplification, the choropleth is useful for achieving insight from data that are otherwise too complex to digest; choropleths increase the amount of information that “can be encoded and subsequently processed or remembered” [2]. The dynamic choropleth offers additional means for both visualization and retrieval of information about data by providing a rich system for filtering and displaying alternative data on maps [6]. Although neuroimaging analyses refer to brain regions, they do not generally treat each region as internally invariant. Because the assumption of internal invariance underpins choropleths, we explore whether insights similar to more conventional neuroimaging analyses can still be achieved with this simplified region-based analysis and how the simplification might facilitate additional insights.

### 1.4 Brain Choropleths

Just like geographic maps, brain maps are generally represented in two dimensions in any given view and contain well-defined regions. Despite the obvious similarities of brain maps and geographic maps, brain choropleths did not seem to be available. Consequently, we created a set of choropleths to provide a tool for the concurrent exploration of brain images and their associated data. In general, the same advantages and disadvantages that apply to using the choropleth for representing statistical data on geographic maps should apply to representing statistical data on brain maps. However, there is an additional consideration: whereas geographic maps are designed to display 2D data, brain maps represent slices through a three dimensional structure, typically viewed in layered axial, coronal and sagittal stacks [7]. To create an appropriate view, we implemented a way to stack choropleths and access them with a slider. This addition to Weave allows the neuroimaging community to take advantage of the advanced tools already available for exploring geographic data.

## Methods

### 2.1 GeoJSON Format

We chose a mapping format, GeoJSON, as the image format for our brain atlas. An alternative image format, the general-purpose scalable vector graphic (SVG), has been used successfully in the Scalable Brain Atlas [8,9]. GeoJSON and SVG are both human readable vector graphics formats well suited to the web. They are based on different data interchange formats: JavaScript Object Notation (JSON) and Extensible Markup Language (XML) respectively, with the former being more compact and faster than the latter [10]. SVG has the advantage, however, of being able to accurately represent curves, whereas GeoJSON must construct all curves from a series of line segments. In addition, most web browsers support SVG directly, whereas GeoJSON is not directly supported by web browsers. Nevertheless, we preferred GeoJSON for several reasons: First, as a mapping format, it is uniquely suited to the representation of layers, coordinates, regional boundaries and associated properties. Second, custom GeoJSON can be readily generated from the original NIfTI format atlases. Third, as the current preferred format for web mapping [11,12] GeoJSON is well suited for leveraging existing geographic visualization tools to display a brain atlas. In comparison, SVG is supported by many image conversion tools and, thus, is better suited for converting images from scans of paper atlases; one of the goals of the Scalable Brain Atlas project. However, SVG is not optimized for mapping and is not supported by Weave. In fact, although most web browsers support SVG directly, the SVG representations used by the Scalable Brain Atlas are not directly supported by web browsers because they depend on a special set of web-based tools and plugins that allow the Scalable Brain Atlas to represent regions as 3D reconstructions, tie the SVG slices together with metadata stored in JSON files, and run PHP scripts to provide web services [8]. Thus, each format has advantages for its particular applications, but GeoJSON was more suitable for our purpose of using existing geographic visualization tools for exploring neuroimaging data.

### 2.2 Generation of GeoJSON files

We developed a set of scripts to convert a 3D FSL atlas into a set of GeoJSON slices. To begin this process, we utilized several FSL functions and general-purpose Unix tools. First, we improved resolution to produce more satisfying regional boundaries (better approximation of curves) by reslicing the atlas into 0.5 mm space before splitting it into slices in each of the three orthogonal directions. We then extracted labels and region indices from the accompanying atlas’s XML file into an ordered list, removed punctuation from the names, and added acronyms for each brain region. Equivalence of brain regions across atlases is imperfect because different atlases subdivide the brain in slightly different ways [13]. We used standardized acronyms (see http://braininfo.rprc.washington.edu) when there was no ambiguity or loss of clarity, and otherwise provided reasonable acronyms. To generate the GeoJSON files we wrote a Matlab function (nifti2geojson.m), which was developed with Matlab 2014a, the Image Processing toolbox, the JSONlab toolbox (http://www.mathworks.com/matlabcentral/fileexchange/33381-jsonlab—a-toolbox-to-encode-decode-json-files-in-matlab-octave) and the NIfTI Image Toolbox (http://www.mathworks.com/matlabcentral/fileexchange/8797-tools-for-NIfTI-and-analyze-image). The nifti2geojson.m function accepts a NIfTI slice in 2 mm, 1mm or 0.5 mm MNI152 space (standard space for the FSL atlases), an ordered region list (the region list we generated previously), and a label indicating the orientation of the current slice (axial, coronal or sagittal). The function then generates a legal GeoJSON file corresponding to that slice. If the slice is axial or coronal, then regions are labeled with the region name and either “_L” or “_R” as determined by the positions of their coordinates. Sagittal slices are labeled as either left or right and every region contained within the sagittal slice is also appropriately labeled. To insure that Matlab could correctly identify the left-right boundary in the NIfTI images, we wrote zeros to the midsagittal slice to represent the hemispheric boundary. Without such a precaution, the processing incorrectly fused some structures (like the frontal pole) across the two hemispheres. As a result of adding the left-right positional labels, there are more “regions” in the GeoJSON slices than in the original XML-extracted region list. We therefore ran another function to extract the case-sensitive region names from the GeoJSON files and output comma-separated values into a file called UniqueRegionList.csv. Crucially, UniqueRegionList.csv identifies each left and right region in the GeoJSON files and is useful for any Weave visualization that uses dynamically linked brain region data [See “Additional Resources” at the end of the paper for a link to an extensive tutorial archive containing the UniqueRegionList.csv and other useful files].

### 2.3 Details of the GeoJSON Slice Files

Each GeoJSON slice file is named with its orientation (“ax”, “cor” or “sag”), slice number, position label (“inf”, “sup”, “post”, “ant”, “L”, “R”) and mm position. For example, ax_156_sup_6mm.geojson is axial slice 156, which is superior to the center axial slice by 6 mm. Positions in mm are preferred because they are shared across atlases at different resolutions, making it easier to find a comparable slice in the 1 mm or 2 mm FSL atlases. Encoded property information for the orientation (a = axial, c = coronal, s = sagittal), position label, and mm position is also stored internal to the GeoJSON file in a hashmap:

{

    "type": "FeatureCollection",

    "orientation": "a",

    "pos": "sup",

    "mm": "6"

    …

}

Within a GeoJSON slice file, each region is defined by the coordinates of its closed polygon boundary and a set of metadata properties, including the short and long region names and the region index:

"properties": {

    "regionName": "FRP_R",

    "regionLongName": "Frontal_Pole_R",

    "regionIdLabel": "1_R",

    "regionId": "1"

}

The GeoJSON slice files were generated from a hybrid of three standard space, population-based atlases of the human brain, including cerebral (cortical and subcortical) and cerebellar regions. The three contributing atlases were the Harvard-Oxford cortical and subcortical structural atlases and the Probabalistic Atlas of the Human Cerebellum (see http://www.cma.mgh.harvard.edu/fsl_atlas.html; HarvardOxford-cort-maxprob-thr25-1mm.nii.gz and HarvardOxford-sub-maxprob-thr25-1mm.nii.gz; [14–16] with permission; Cerebellum-MNIflirt-maxprob-thr25-1mm.nii.gz; [17,18] in accordance with the Creative Commons Attribution-NonCommercial Unported License). We chose linear, rather than non-linear, alignment for the cerebellar atlas to remain consistent with the alignment of the two cerebral atlases. Note that regional indices in our resulting hybrid atlas do not always correspond to the original indices in the three contributing atlases because of overlapping number schemes.

To provide a balance between the total number of GeoJSON files and a good selection of slices through various brain regions, we generated display slices of interest every 6 mm in axial, coronal and sagittal orientations. Our GeoJSON brain map files are freely available here: https://sites.google.com/a/email.arizona.edu/brain_choropleths/ along with the 0.5 mm hybrid atlas in NIfTI format (from which the GeoJSON files were derived), and an archive of extensive tutorial materials that allow interested researchers to examine each of the four unlocked visualizations described in this paper, and build them from scratch using the original data [See “Additional Resources” at the end of this paper]. All scripts and files available from the website are made available in accordance with the Creative Commons Attribution-NonCommercial Unported License https://creativecommons.org/licenses/by-nc/3.0/deed.en_GB, unless they are already protected by an alternative license.

## Results and Discussion

To demonstrate the utility of the Weave workbench and the simplified region-based analysis for developing insights into data, we have constructed four visualizations that represent different views onto a specific dataset. Each visualization is motivated by particular questions and optimized to explore those questions. Because the data have already been analyzed with a more traditional approach [19], we can verify that the Weave-based analysis offers comparable insights, but that it also permits us to develop novel insights.

The fMRI experiment we analyze explored the initial stages of language learning in adults exposed to a novel language, Icelandic, over the course of four consecutive scans [19]. All subjects in the original study were consented in accordance with the University of Arizona IRB. The data were analyzed using independent component analysis. Each independent component (IC) represents a distinct source signal, and regions that activated significantly with this signal were said to be functionally connected even if they were not physically contiguous. Although ICs varied in location and intensity across scans and participants, the ICA analysis software insured that all scans and participants displayed the same set of ICs. We considered activation differences between ICs in a single scan, for a given IC across scans, and in two groups who were high or low performing on the learning task. This type of data, which includes multiple signals for each scan, multiple scans within an experiment, and subgroupings of participants, poses a significant visualization challenge.

In the original study, we were interested in task-related ICs: ICs that were correlated to the listening phase, the test phase, or both. All four ICs we identified were correlated to listening more than control; and IC-1, 2 and 4 were correlated to test more than listening. That original analysis revealed strikingly different patterns of activation across scans in the four different task-related ICs. Specifically, we found that IC-1 and IC-2 were more active early in the experiment (scans 1 or 2), but IC-3 and IC-4 were more active later in the experiment. Interestingly, we found greater group average signal change in low performers than in high performers, and group average signal change was always negatively correlated to success at the task suggesting that “greater physiological effort was expended” by the low performers. In addition, the original analysis revealed that two ICs were significantly lateralized: IC-2 was left-lateralized and IC-3 was right-lateralized. [19]

The original and current analyses differ in their approach to identifying areas of significant activation. In the original analysis, IC activations were not constrained or divided by regional boundaries (other than the left and right hemispheres); each voxel that met criterion (group-level t-stat at p < .05 uncorrected) was also required to belong to a cluster of sufficient size so as to indicate non-chance activation in a brain-wide analysis [19]. By contrast, in the simplified region-based analysis that underpins the choropleths, IC activations were divided by regional boundaries defined by the cerebral and cerebellar atlases. No formal statistical correction was applied in the region-based analysis; rather, we retained a region and its associated value if that region’s mean t-stat met criterion (p < .05 uncorrected). This meant that a region could be included despite containing some subthreshold voxels, but also that significant voxels in a region of low overall activation would not be included in the analysis. This region-aware criterion takes into account both the size and location of the activations. For example, the size of suprathreshold activation necessary to reach criterion is different for small regions (e.g., subcortical or cerebellar) than for large regions (e.g., frontal). In addition, only suprathreshold activations located in grey matter are considered. Despite the differences, the net effect of the simplified region-based analysis was similar to the original correction based on cluster size.

In the current analysis, we prepared group and individual level data for Weave by extracting mean values for each standard-space region. At the group level, we retained mean t-stat values for each region that met criterion. At the individual participant level, we extracted mean percent signal change, and retained data for each individual only in regions with a positive signal change and suprathreshold group t-stat. These thresholding choices allowed us to stay as close as possible to the original analysis so that the primary difference between the two analyses was the choice to look at brain-wide activation or to chunk activation by regions. A crucial question of this research was whether the current simplified region-based analysis could be used to derive the same sorts of insights as the original analysis, despite the different approaches to quantifying regional activation.

### 3.1 Visualization 1

**Fig 1:**
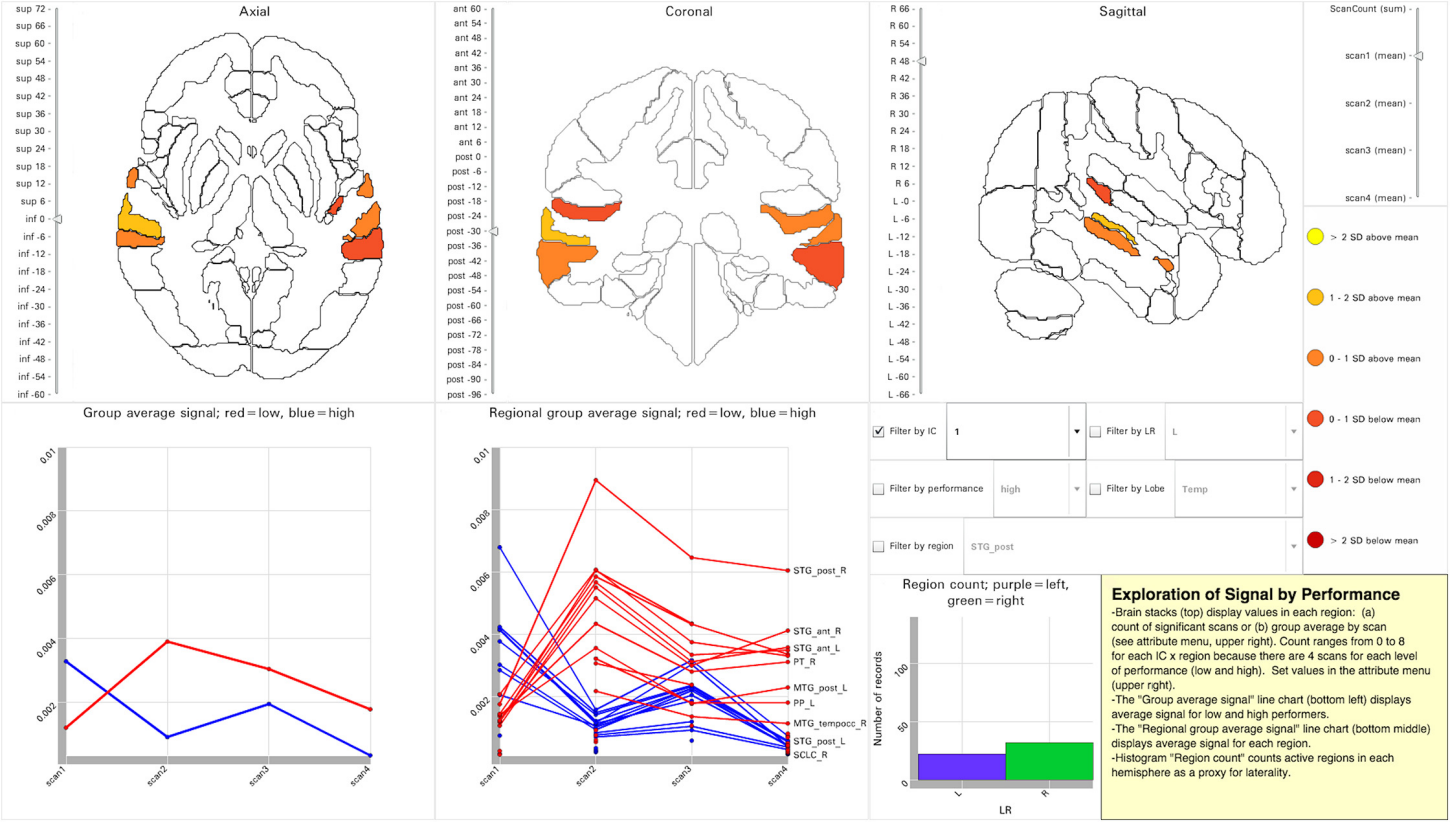
Tool Layout for Visualization 1. http://demo.oicweave.org/weave.html?file=brain-choropleths/visualization1.weave. Mean regional activation for IC-1 during scan 1 is displayed in the brain maps. IC-1 was selected from the set of filters (middle right). Scan 1 was selected from the attribute menu (upper right). In the lower half of the figure, two line charts show mean activation intensity at each scan for high (blue) and low (red) performers: group average (bottom left) and regional average (bottom middle). The purple and green histogram (bottom middle) provides laterality information by comparing the count of active regions in the left hemisphere to that in the right hemisphere.

**Fig 2:**
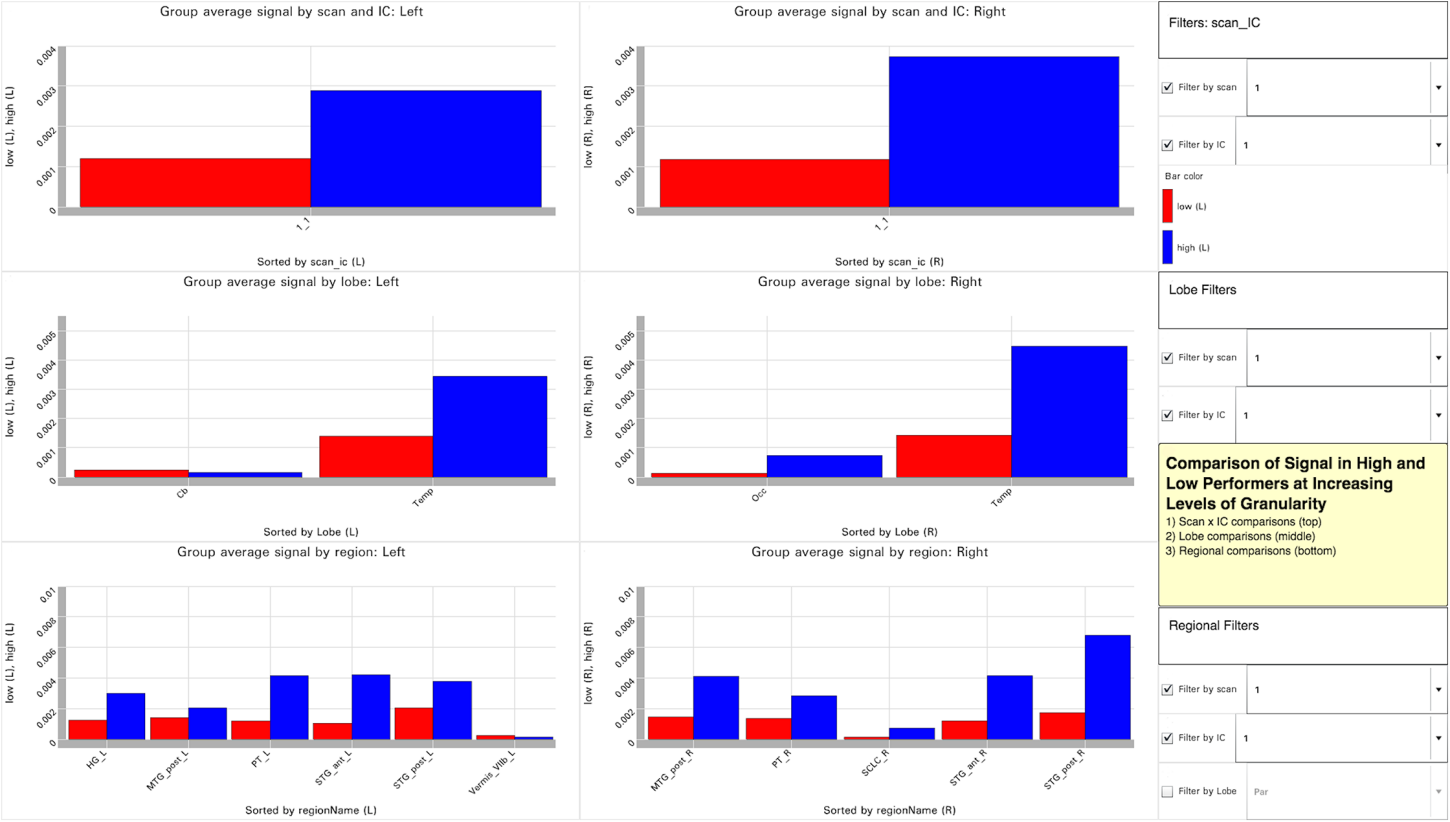
Tool Layout for Visualization 2. http://demo.oicweave.org/weave.html?file=brain-choropleths/visualization2.weave. This visualization is designed to contrast subgroups of subjects, in this case high and low performers, on the behavioral task used during the scan. The figure displays data for IC-1 at scan 1 (see filters to the right of each bar chart). The bar charts are arranged at increasing levels of granularity from top to bottom and divided into left and right hemisphere activation. The top row of charts displays mean signal intensity for scan 1 and IC-1 by performance. The second row further subdivides the signal by lobe, and the bottom row compares regional values.

Visualization 1 is a region-based dynamic visualization (Fig 1; http://demo.oicweave.org/weave.html?file=brain-choropleths/visualization1.weave) modeled after Fig 2 from the original analysis [19]. The visualization is built on two tables with records related by regionName. One table, the UniqueRegionList, contains all 146 brain regions and their properties. The second table contains a detailed record for each region that has suprathreshold activation for at least one IC and scan. The underlying data can be explored in Weave by choosing “Data → Manage or browse data”, or by choosing “Tools → + Table” and selecting the fields to be displayed (see “Additional Resources” at the end of this paper for links to the Weave tutorial). Data filters can limit the displayed records by IC, hemisphere (LR), performance, lobe, or region. Like the original figure [19], Visualization 1 displays brain images and line charts of signal change.

Compared to the brain renderings in the original static figure from [19], the brain choropleths in Visualization 1 present a variety of opportunities to explore the underlying data. The figure from the original paper displayed four brain renderings for each IC: lateral and medial, left and right views. Each IC was represented with a different color scale, and each color scale included seven levels: four to represent the presence of significant activation in scans 1, 2, 3, 4 and three to represent overlap in multiple scans (overlap of two, three or four scans). By contrast, because the choropleths do not need to represent all the data at once, they both simplify the data presentation and provide more detail than the 3D renderings. For example, a single color scale based on standard deviations unifies all of the mapped data, and because only one attribute is displayed at a time, the color scale can be used to represent signal intensity or overlap. The attribute to be displayed is chosen from a slider (upper right) that separates the display of scan count from the display of group average signal for each scan. The binning and the color scale can be altered by choosing “Tools → Color Controller” from the menu at the top of the Weave interface.

Because the choropleths are organized into three orthogonal stacks of brain slices, the user can examine all regions, including those deep within the brain, and not just rendered surfaces. A vertical slider next to each brain stack allows the user to set the displayed slice by position in millimeters. Because the choropleth stacks display a single slice at a time in each orientation, they fall short of the 3D renderings in their ability to provide an immediate appreciation of laterality. However, a histogram (bottom middle) quantifies laterality by displaying the count of suprathreshold regions on the left and right. Enabling and disabling the filters (lower right quadrant) offers additional potential for data exploration; for example, group average signal at each scan can be displayed separately for high and low performers by setting the performance filter. Choosing “ScanCount” in the attribute menu tool displays a count of how often a region is activated (maximum 32: 4 ICs x 4 scans x 2 performance levels). While scan count is selected, disabling all filters reveals regions with the most frequent suprathreshold activation irrespective of IC or scan.

**Fig 3:**
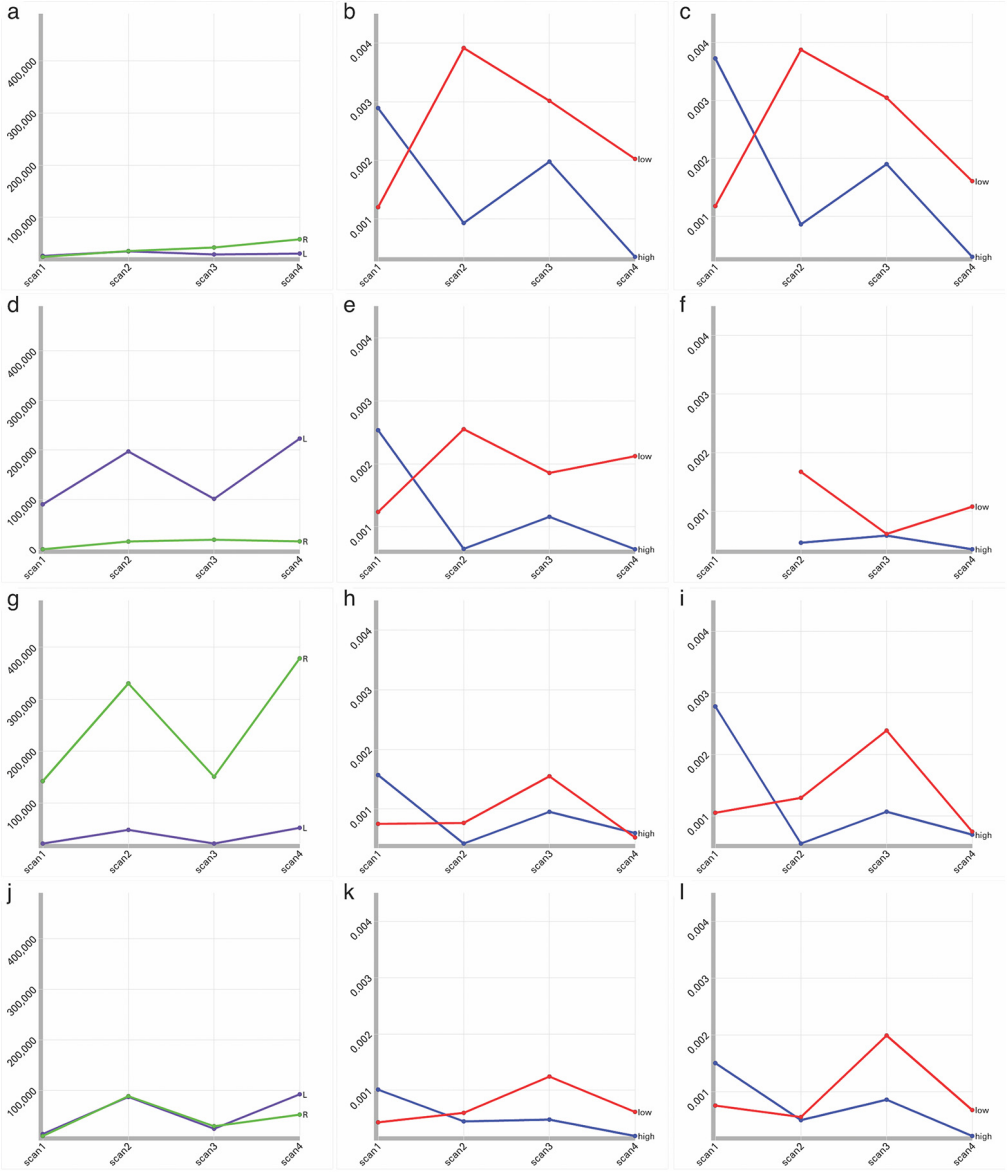
Line charts of Activation Extent and Intensity. Each row represents a component (IC-1 (a-c), IC-2 (d-f), IC-3 (g-i) and IC-4 (j-l)). The first column represents changes in the extent of significant activation for the IC on the left (purple) and right (green) from scan 1 through 4. The second (left hemisphere) and third (right hemisphere) columns compare group average percent signal change for each IC in low performers (red) and high performers (blue) from scans 1 through 4. The figure reveals that there is no clear relationship between volume variation (column 1) and signal intensity (columns 2 and 3). The figure also demonstrates that signal intensity variation in this simplified region-based analysis is comparable to signal intensity variation in the more traditional analysis.

Whereas the brain maps display regional data for either the scan count or the signal during a scan, the line charts display group average signal change across all four scans. On the lower left the “group average signal” line chart compares high and low performers across the four scans and averaged across regions. This is very similar to the line charts in Fig 2 of the original analysis [19]. Although this division reflects the participant subgroups used in the original study, other participant subgroupings (e.g., normal vs. impaired, +genotype vs.–genotype, young vs. old) could be displayed in this manner. By applying the IC and LR filters, this dynamic line chart can be configured to display lines corresponding to the eight line charts in the original Fig 2 [19] (sans standard error bars) [See Fig 3]. The “regional group average signal” line chart supplements the “group average line chart” by comparing high and low performers across the four scans for each region. We can drill down into this chart to reveal novel patterns. For example, disabling all filters reveals exceptionally high regional signal in the right posterior superior temporal gyrus in low performers. Filtering by IC reveals that this signal is associated with IC-1. Filtering by region (e.g., STG_post) allows us to compare this exceptional signal in high and low performers to the homotopic region in the contralateral hemisphere. Given the role of the right posterior superior temporal gyrus in processing human voice [20], we can reasonably conclude that attention to human voice was an especially active focus of brain resources throughout the experiment.

This simplified region-based analysis produced results comparable to the original analysis. The activation patterns represented by the lines on the “group average signal” chart were extremely similar to the line charts in Fig 2 of the original paper [19]. High performers showed reduced activation after scan 1, whereas low performers showed increased activation after scan 1. In addition, lateralization patterns reported in the original analysis for each IC were retained by the current analysis (the IC filter and the left-right histogram can be used to verify the patterns; see Fig 3). Some minor differences from the original analysis did occur, especially for IC-2: For scan 1 on the right, no regions survived our region-based criterion for inclusion in the choropleth maps, whereas the original analysis found some significant activation. Also for IC-2, the signal in scan 3 on the left was higher for the low performers than in the original analysis, although the pattern of rise and fall across the scans was similar (signal increased for scans 2 and 4 compared to scans 1 and 3). Despite being slightly more conservative than the original analysis, the simplified region-based approach was generally comparable.

In addition to replicating the results of the more traditional analysis, the dynamic nature of the visualization facilitated novel insights. For example, by disabling all filters and setting the attribute menu to “Scan Count”, the choropleths revealed the most frequently activated regions in the brain across scans and ICs. These include the middle temporal gyri, which have frequently been reported in word segmentation studies [19]; the supramarginal gyri, implicated in phonological decision making [21]; and the paracingulate gyrus, implicated in action monitoring and awareness of errors [22, 23]. The frequent activation of these regions suggests that they may be hubs supporting the interaction of multiple ICs.

Visualization 1 demonstrates that the use of choropleths, and the associated simplification of intensity variation, did not significantly alter our ability to derive insights comparable to the original analysis. In fact, the simplification inherent in choropleths could facilitate novel insights for both the original researcher and readers with different agendas. Visualizations 2, 3 and 4 explore additional information available with alternative presentations of the data. Although choropleths were not explicitly used in these additional visualizations, all visualizations depended on the same underlying quantization of the data at the regional level.

### 3.2 Visualization 2

We designed Visualization 2 (Fig 2; http://demo.oicweave.org/weave.html?file=brain-choropleths/visualization2.weave) to facilitate comparison of high and low performers across regions and lobes in a single visual snapshot. The choropleths in Visualization 1 did not allow the user to see all regions at once or to view multiple values in a region at one time. Visualization 2 is built on three independent tables that compare mean signal in high and low performers. The data in each table is displayed as a pair of bar charts for the left and right, respectively. The top row corresponds to the first table (31 records) and groups signal by scan and IC. The middle row corresponds to the second table (97 records) and groups signal by scan and IC in each lobe. The bottom row corresponds to the third table (279 records) and groups signal by scan and IC in each region. Each pair of bar charts is controlled by the scan and IC filters directly to their right. An additional lobe filter for the bottom pair of bar charts can be used to limit the overcrowding of regional bars. This visualization is intended to be used by setting all three sets of filters to the same values, so as to compare high and low performers at increasing levels of granularity from top to bottom.

Visualization 2 facilitated the novel insight that regions and lobes behave quite differently from one another and that the simple division between left and right in the original paper obscured some potentially interesting patterns. For example, disabling the scan filter for performance by lobe (the middle row) and setting the IC filter to “1” reveals that the IC-1 line chart in the original analysis results primarily from the high signal and large performance-based differences in posterior temporal lobe structures. Such large values obscured the fact (now visible in Visualization 2) that most regions of the brain displayed low signal and small performance-based differences for IC-1. IC-2 displays the opposite pattern, high signal and large performance-based differences occurred primarily in frontal and parietal but not temporal regions. Visualization 2 also identifies frequent activation of the nucleus accumbens for both IC-2 and IC-4, consistent with the possibility that these components are involved in reward and motivation networks [24].

In Visualizations 1 and 2 we saw two different patterns that are commonly examined in fMRI analyses: one involving changes in signal intensity, and the other involving changes in extent. However, neither of those visualizations was optimized for examining extent or determining whether intensity and extent were related. Additionally, our simplified region-based analysis naturally treats large and small regions as having the same weight. Consequently, we developed a different Weave visualization to overcome these issues.

### 3.3 Visualization 3

**Fig 4:**
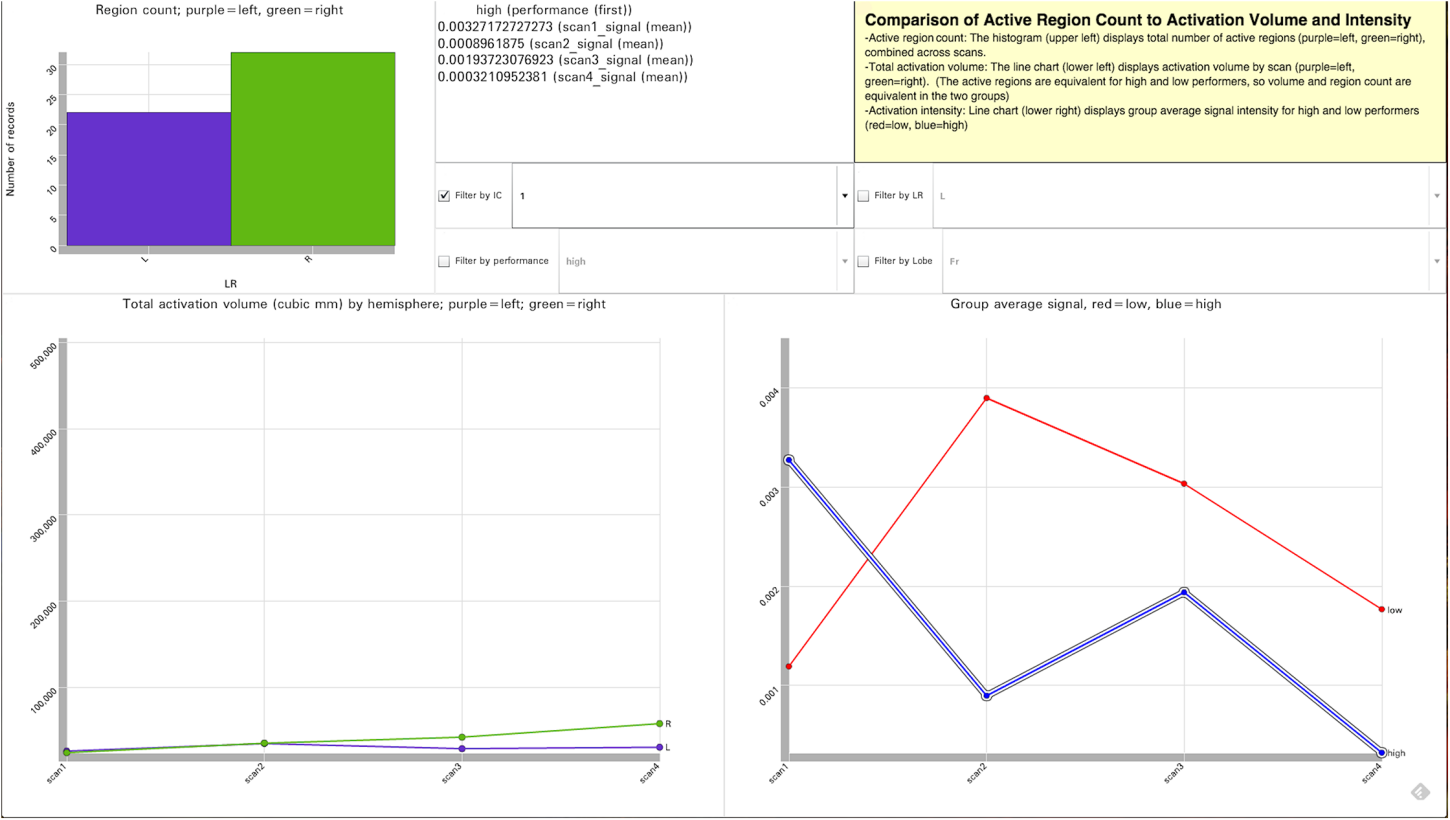
Tool Layout for Visualization 3. http://demo.oicweave.org/weave.html?file=brain-choropleths/visualization3.weave. This visualization was designed to explore a novel question about the relationship between signal intensity and extent. To simplify the current discussion, the figure displays the visualization filtered by IC-1. The data could as easily be filtered by any of the four ICs. Three different metrics of the activations are compared: the number of suprathreshold regions by hemisphere for all scans (histogram, upper left), the total activation volume by scan for each hemisphere (lower left line chart), and group average intensity of the activations by scan for high and low performers (lower right line chart). Because high and low performers activate the same regions, there is no difference in number of regions or volume of regions by performance. Four filters allow the user to limit the dataset by IC, performance, hemisphere (LR) and lobe. The numerical values for the highlighted blue line (lower right of figure) are displayed in the dynamic mouseover window (top middle).

Visualization 3 (Fig 4, http://demo.oicweave.org/weave.html?file=brain-choropleths/visualization3.weave) was created to explore a novel question about the relationship between signal intensity and extent (i.e., volume of activation). Using Visualization 3 we can ask, for example, whether intensity and extent rise and fall together or in opposition, or whether they are unrelated. We can also examine more complex relationships with relative ease; e.g., does a given pattern exist primarily in a subset of the lobes? Visualization 3 is based on the same 268 records used in Visualization 1 but each record has four additional fields which contain the regional volume if the region is suprathreshold during that scan.

The line chart on the left “Suprathreshold volume in cubic mm by hemisphere” compares left and right suprathreshold volumes at each scan. Because the suprathreshold regions were based on group level t-stats irrespective of performance, just as they were in the original analysis, volume variation does not differ by performance. Above the volume graph is a histogram “Region count” indicating the number of records with suprathreshold regions on the left (purple) and right (green). This makes it possible to compare volume variation to overall regional counts. If it were the case that small regions behaved very differently than large regions, then total volume and region count might display different patterns. The line chart on the bottom right is equivalent to the graph of the same name in Visualization 1 and displays the group average signal for high and low performers. Filters can limit the records by IC, performance, hemisphere (LR) or lobe.

Visualization 3 revealed that activation extent did not necessarily mirror signal intensity; thus extent and signal intensity analyses offer distinct insights [See Fig 3]. For example, despite displaying very little variation in volume from scan to scan, IC-1 displayed high signal intensity changes. In contrast, the other three ICs displayed a pattern of volumetric expansion and contraction from scan to scan (increased volume in scans 2 and 4 relative to scans 1 and 3). Again, variations in activation extent did not have a clear relationship to the variation in signal intensity (Fig 3). Filtering by lobe suggested that the volumetric variation was primarily attributable to changes in the frontal lobes. Specifically, the patterns of volumetric expansion and contraction in IC-2, 3 and 4 all occurred primarily in frontal lobe; whereas IC-1, which had no suprathreshold activation in the frontal lobe, exhibited little volumetric variation.

Because the focus of visualizations 1, 2 and 3 was to compare high and low performers, it was difficult to determine at a glance which regions were consistently suprathreshold across scans or in multiple ICs at once. For example, because of the performance-based comparison goal, the “group average by region” line chart in Visualization 1 contained twice as many lines as were necessary for answering this kind of continuity question. Visualization 2 facilitated exploration of scan snapshots but not patterns across scans. Visualization 3 provided average brain-wide or lobe values across scans and was, thus, unsuitable for looking at continuity of regional activation from scan to scan.

### 3.4 Visualization 4

**Fig 5:**
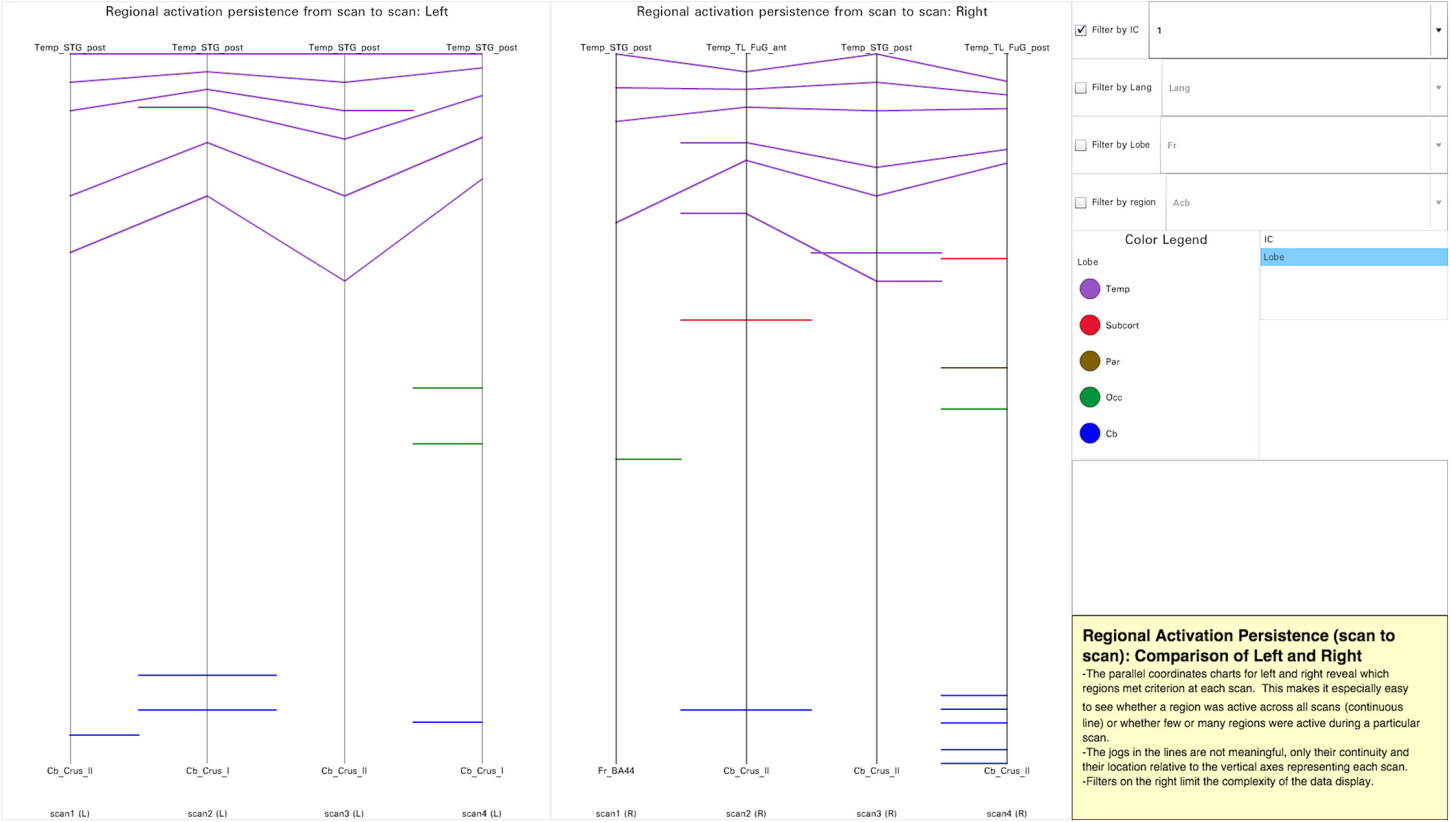
Tool Layout for Visualization 4. http://demo.oicweave.org/weave.html?file=brain-choropleths/visualization4.weave. This visualization was designed to display the continuity of activation for each region from one scan to the next. For purposes of this discussion, the data is filtered to show only data for IC-1. Lines are colored by lobe in the figure, but the attribute menu to the right of the color legend allows the user to color the lines by IC. It is apparent that IC-1 has a lot of suprathreshold regions in the temporal lobe (purple) and most of the activations reoccur in all four scans.

Visualization 4 (Fig 5, http://demo.oicweave.org/weave.html?file=brain-choropleths/visualization4.weave) was developed to compare how continuously regions are activated across the scans. This is the only one of our visualizations that does not separate signals based on performance. In this visualization, we can see at a glance whether a region comes online briefly or persistently and, at any scan, how many regions are online. This is particularly useful for data about learning collected over multiple scans in a single session, and could be equally useful for longitudinal studies where scans are collected across longer time frames. Visualization 4 is based on a single table of 134 records. Each record names a region that was suprathreshold in one or more scans and indicates which scans were suprathreshold (mean t-stat above threshold in the group). Regions are named so that the lines will be sorted by lobe (e.g., Temp_STG_post for posterior superior temporal gyrus).

Visualization 4 is implemented with two parallel coordinates charts (left and right hemisphere). Rather than containing the usual orthogonal x and y-axes, parallel coordinates charts are composed of parallel axes each representing a list of categories or dimensions. In Visualization 4 each vertical axis represents a scan. Each line that crosses one of these four axes represents a region that is suprathreshold at that scan. The spacing along the vertical dimension of the chart carries no meaning and only serves to separate the regions so that they can be easily viewed. Likewise, line slant is not informative. Rather, information resides in line continuity, color, and grouping by lobe along the vertical axes. A line that is continuous from scans 1 through 4 indicates a region that was persistently active in that IC in all four scans. Discontinuous lines indicate regions only active for three or fewer scans, as reflected by which vertical axes are crossed. This display makes it very easy to appreciate which regions were consistently activated and which activated only during certain periods (scans) of the experiment. Although here the axes represent scans within an experiment, one could easily utilize the same type of display to show consistency of regional activation in a meta-analytic approach across similar studies.

The lines of the parallel coordinates charts are grouped alphabetically by lobe and colored by IC or lobe (set in the attribute menu to the right of the color legend). If the lines are colored by IC and a region is active for multiple ICs, only one color will be visible; however, by probing with a mouse over the line, all ICs associated with the line will be highlighted in the color legend. In addition, probing over a line displays the region name and highlights the line even if it is discontinuous. Selecting an IC in the color legend will highlight all lines associated with that IC. Filters limit the density of the displayed information by IC, “Lang” (whether or not a brain region is classically associated with language), lobe and region.

The parallel coordinates charts indicate that while some regions were consistently active throughout the experiment, others were only active transiently. This is consistent with the data reported in the original study [19]. In addition, this visualization reveals several novel types of information at a glance. For example, persistently suprathreshold regions appear mostly in the temporal lobe for IC-1, entirely on the left for IC-2, and in large numbers on the right for IC-3. In addition, some regions come online only for a short time, especially during scans 2 and 4. Drilling down into the visualization allows us to look for more complex patterns involving multiple regions. For example, the middle frontal gyri (a.k.a. dorsolateral prefrontal cortex) and frontal poles are known to be involved in processing integrated information in working memory, with the middle frontal gyri being more sensitive to task complexity [25]. IC-2 (on the left) and IC-3 (on the right) display persistent activation in the middle frontal gyri, and during scans 2 and 4 these two ICs also display activation in the left and right frontal pole respectively, consistent with their involvement in information integration. IC-4 displayed persistent suprathreshold activity in regions associated with motor activity (left supplementary motor cortex), as well as attention and motivation (anterior cingulate) [26], consistent with it being involved in button pressing during the test phase.

As shown in Fig 5, the lines can also be colored by lobe. This allows us to examine the co-occurrence of suprathreshold activation in different lobes more easily: using the attribute menu to the right of the color legend, switch color data from “IC” to “Lobe”. Turn on the “Lang” filter and choose “Lang” rather than “Non”. These settings facilitate examination of co-occuring suprathreshold activity in frontal and temporal language areas. If, as Marslen-Wilson and colleagues suggest [27], it is the connection between temporal and frontal language regions that accounts for syntactic processing, then ICs that display suprathreshold activation in both areas simultaneously (IC-2 and, to a lesser extent, IC-4) are candidates for syntactic processing.

### 3.5 Summary

We prepared four Weave visualizations to illustrate our points. We demonstrated that a simplified region-based analysis can provide results comparable to our original analysis and that Weave visualizations can be used to facilitate additional insight into this same dataset.

## Conclusion

All visualizations involve making choices about what information to sacrifice and what to display. Certainly a simplified region-based analysis suitable for viewing with choropleths sacrifices some details of the data, most notably the locations of activation peaks within specific brain regions. An advantage of the simplified region-based approach is that the data can easily be examined using choropleths and related dynamic visualizations. In this paper we have demonstrated that robust findings survive in this simplified region-based analysis. Furthermore, we have demonstrated the flexibility of this approach for exploring multiple aspects of the data that would be difficult to visualize with more traditional brain image displays.

Weave is intended to supplement rather than replace existing methods. For example, Weave is a workbench for dynamically exploring data, it is not a graphics creation tool. Using Weave and region-based analyses does not prevent one from working with other approaches; for example, reporting x-y-z locations of activation peaks in tabular form. Weave is also an evolving set of tools that requires some effort to learn. We have provided extensive tutorial materials to help with the process [see “Additional Resources” at the end of this paper], and Weave maintains an active forum: https://groups.google.com/forum/-!forum/weave-users. For those who are willing to face the learning curve, Weave visualizations provide considerable flexibility in data exploration. Although it may take some time initially to learn to set up the more complex Weave visualizations, once a visualization is set up, it is possible to print graphics, replay the history of our data exploration, switch to different datasets, and make the visualization available on the web. The ability to switch datasets is especially useful because projects often comprise a set of related experiments. By reusing the Weave visualizations, analysis of related experiments becomes easier and more consistent. In addition, if we choose to make visualizations available on the web (for example, as supplements to journal articles) the Weave web application offers granular control over which visualization features are publicly available. For instance, Weave allows the researcher to restrict access to tool settings, additional tools, underlying data, export and save features, to name just a few.

### 4.1 Future Directions

We believe that neuroinformatics will benefit from expanding its focus to include better ways to represent the associated data (behavioral, genetic, medical, etc.) that often accompanies brain images. The choropleth atlas developed here, and used in conjunction with the Weave visualization environment, is a step in that direction. The choropleths presented here are based on anatomically defined grey matter regions, but future choropleth atlases could be based on different regions, such as functional regional boundaries, which might be more appropriate for other types of studies [28]. In contrast to choropleths, more traditional types of brain displays (e.g., brain renderings and yoked orthogonal viewers) have already been implemented in several web applications [1]. Adding support to Weave for these types of displays could prove highly beneficial to neuroimaging research. Finally, we hope that our modest contribution to data visualization for neuroinformatics will inspire others to investigate novel ways of representing the complex data associated with the brain, and to find ways to make that data easier to explore, analyze, discuss, and share.

## Additional Resources

An extensive illustrated tutorial kit is available here: https://sites.google.com/a/email.arizona.edu/brain_choropleths/

Download WeaveTutorial.zip (bottom of web page). The archive contains a step-by-step illustrated tutorial pdf and all necessary data tables for creating each of the four visualizations discussed in this paper. In addition, the archive contains unlocked copies of all four finished visualizations for the reader to modify and explore at will. Other resources can be downloaded from this same site: the GeoJSON slices, the original NIfTI brain atlas, and all the scripts for creating GeoJSON files from NIfTI atlases.

## References

[1] Sherif T, Kassis N, Rousseau M-É, Adalat R, Evans AC. BrainBrowser: distributed, web-based neurological data visualization. Frontiers in Neuroinformatics. Frontiers; 2015;8 10.3389/fninf.2014.00089 PMC4292582 25628562

[2] Patterson RE, Blaha LM, Liggett KK. A human cognition framework for information visualization. Computers & Graphics. 2014;42: 42–58.

[3] Baumann A, Dufilie AS, Kolman S, Kota S, Mass W. Exploratory to Presentation Visualization, and Everything In-between: Providing Flexibility in Aesthetics, Interactions and Visual Layering. IEEE; 2011 pp. 200–204. 10.1109/IV.2011.82

[4] Boscoe FP, Pickle LW. Choosing geographic units for choropleth rate maps, with an emphasis on public health applications. Cartography and Geographic Information Science. 2003 10.1559/152304003100011171

[5] Friendly M, Denis DJ. Milestones in the history of thematic cartography, statistical graphics, and data visualization. In: Michael Friendly [Internet]. 2008 [cited 23 Sep 2014]. Available: http://euclid.psych.yorku.ca/SCS/Gallery/milestone/milestone.pdf

[6] Norman K, Zhao H, Shneiderman B. Dynamic query choropleth maps for information seeking and decision making. In: http://hcil.cs.umd.edu/trs/2003-23.html [Internet]. 2003 [cited 22 Sep 2014]. Available: http://hcil2.cs.umd.edu/trs/2003-23/2003-23.html

[7] Maye A, Wenckebach TH, Hege HC. Visualization, reconstruction, and integration of neuronal structures in digital brain atlases. Int J Neurosci. 2006;116: 431–459. 10.1080/00207450500505860 16574581

[8] Bakker R, Tiesinga P, Kotter RK. The Scalable Brain Atlas: instant web-based access to public brain atlases and related content. CORD Conference Proceedings. 2013. 10.1007/s12021-014-9258-x PMC4469098 25682754

[9] Majka P, Kublik E, Furga G, Wójcik DK. Common atlas format and 3D brain atlas reconstructor: infrastructure for constructing 3D brain atlases. Neuroinform. 2012;10: 181–197. 10.1007/s12021-011-9138-6 PMC3325030 22227717

[10] Nurseitov N, Paulson M, Reynolds R, Izurieta C. Comparison of JSON and XML Data Interchange Formats: A Case Study. CAINE. 2009;: 157–162.

[11] Butler H, Daly M, Doyle A, Gillies S, Schaub T. The GeoJSON format specification. In: http://www.geojson.org/geojson-spec.html [Internet]. 2008 [cited 4 Oct 2014]. Available: http://www.geojson.org/geojson-spec.html

[12] Bostock M. Code as cartography. The Cartographic Journal. 2013;50: 129–135. 10.1179/0008704113Z.00000000078

[13] Bohland JW, Bokil H, Allen CB, Mitra PP. The Brain Atlas Concordance Problem: Quantitative Comparison of Anatomical Parcellations. Sporns O, editor. PLoS ONE. 2009;4: e7200 10.1371/journal.pone.0007200 19787067 PMC2748707

[14] Frazier JA, Chiu S, Breeze JL, Makris N, Lange N, Kennedy DN, et al Structural Brain Magnetic Resonance Imaging of Limbic and Thalamic Volumes in Pediatric Bipolar Disorder. American Journal of Psychiatry. American Psychiatric Association; 2005;162: 1256–1265. 10.1176/appi.ajp.162.7.1256 15994707

[15] Goldstein JM, Seidman LJ, Makris N, Ahern T, O’Brien LM, Caviness VS Jr, et al Hypothalamic Abnormalities in Schizophrenia: Sex Effects and Genetic Vulnerability. Biological Psychiatry. 2007;61: 935–945. 10.1016/j.biopsych.2006.06.027 17046727

[16] Makris N, Goldstein JM, Kennedy D, Hodge SM, Caviness VS, Faraone SV, et al Decreased volume of left and total anterior insular lobule in schizophrenia. Schizophrenia Research. 2006;83: 155–171. 10.1016/j.schres.2005.11.020 16448806

[17] Diedrichsen J, Balsters JH, Flavell J, Cussans E, Ramnani N. A probabilistic MR atlas of the human cerebellum. NeuroImage. 2009;46: 39–46. 10.1016/j.neuroimage.2009.01.045 19457380

[18] Diedrichsen J, Maderwald S, Küper M, Thürling M, Rabe K, Gizewski ER, et al Imaging the deep cerebellar nuclei: A probabilistic atlas and normalization procedure. NeuroImage. 2011;54: 1786–1794. 10.1016/j.neuroimage.2010.10.035 20965257

[19] Plante E, Patterson DK, Dailey NS, Almryde K, Fridriksson J. Dynamic changes in network activations characterize early learning of a natural language. Neuropsychologia. Elsevier; 2014;62: 77–86. 10.1016/j.neuropsychologia.2014.07.007 25058056 PMC4167491

[20] Mattschey J, Sammler D, Belin P, Anwander A. Anatomical Connectivity of Temporal Voice Areas. BNA Neuroscience Festival. 2015 pp. 309–312.

[21] Hartwigsen G, Baumgaertner A, Price CJ, Koehnke M, Ulmer S, Siebner HR. Phonological decisions require both the left and right supramarginal gyri. Proceedings of the National Academy of Sciences of the United States of America. 2010;107: 16494–16499. 10.1073/pnas.1008121107 20807747 PMC2944751

[22] Veen VV, Carter CS. The Timing of Action-Monitoring Processes in the Anterior Cingulate Cortex. J Cogn Neurosci. 2002;14: 593–602. 10.1162/08989290260045837 12126500

[23] Amodio DM. Meeting of minds: the medial frontal cortex and social cognition. Nat Rev Neurosci. 2006;7: 268–277. 10.1038/nrn1884 16552413

[24] Knutson B, Adams CM, Fong GW, Hommer D. Anticipation of increasing monetary reward selectively recruits nucleus accumbens. J Neurosci. 2001;21: RC159–RC159. 11459880 10.1523/JNEUROSCI.21-16-j0002.2001 PMC6763187

[25] Kim C, Kroger JK, Calhoun VD, Clark VP. The role of the frontopolar cortex in manipulation of integrated information in working memory. Neuroscience Letters. 2015;595: 25–29. 10.1016/j.neulet.2015.03.044 25818331 PMC4495662

[26] Binder JR, Desai RH, Graves W, Conant L. Where Is the Semantic System? A Critical Review and Meta-Analysis of 120 Functional Neuroimaging Studies. Cereb Cortex. 2009;19: 2767 10.1093/cercor/bhp055 19329570 PMC2774390

[27] Marslen-Wilson WD, Stamatakis EA, Tyler LK. Functional Organization of the Neural Language System: Dorsal and Ventral Pathways Are Critical for Syntax. Cereb Cortex. 2012;23: 139–147. 10.1093/cercor/bhr386 22275482 PMC3601415

[28] Shirer WR, Ryali S, Rykhlevskaia E, Menon V, Greicius MD. Decoding Subject-Driven Cognitive States with Whole-Brain Connectivity Patterns. Cereb Cortex. 2011. 10.1093/cercor/bhr099 PMC3236795 21616982

[29] Aigouy B, Mirouse V. ScientiFig: a tool to build publication-ready scientific figures. Nature Methods. Nature Publishing Group; 2013;10: 1048–1048. 10.1038/nmeth.2692 24173380

